# Intra- and inter-examination reproducibility of T2 mapping for temporomandibular joint assessment at 3.0 T

**DOI:** 10.1038/s41598-022-15184-9

**Published:** 2022-06-29

**Authors:** Pongsapak Wongratwanich, Toshikazu Nagasaki, Kiichi Shimabukuro, Masaru Konishi, Masahiko Ohtsuka, Yoshikazu Suei, Takashi Nakamoto, Yuji Akiyama, Kazuo Awai, Naoya Kakimoto

**Affiliations:** 1 Department of Oral and Maxillofacial Radiology, Graduate School of Biomedical and Health Sciences, Hiroshima University, 1-2-3 Kasumi, Minami-ku, Hiroshima, 734-8553 Japan; 2 Department of Oral and Maxillofacial Radiology, Hiroshima University Hospital, 1-2-3 Kasumi, Minami-ku, Hiroshima, 734-8553 Japan; 3 Department of Radiology, Hiroshima University Hospital, 1-2-3 Kasumi, Minami-ku, Hiroshima, 734-8553 Japan; 4 Department of Diagnostic Radiology, Graduate School of Biomedical and Health Sciences, Hiroshima University, 1-2-3 Kasumi, Minami-ku, Hiroshima, 734-8553 Japan

**Keywords:** Musculoskeletal system, Cartilage, Imaging techniques

## Abstract

T2 mapping allows quantification of the temporomandibular joint (TMJ) ultrastructural degeneration. The study aimed to assess intra- and inter-examination reproducibility of T2 mapping for TMJ evaluation at 3.0 Tesla (T). Seventeen volunteers, regardless of temporomandibular disorder (TMD) diagnosis, received magnetic resonance (MR) examination at 3.0 T. T2 mapping was performed twice (> 5 min between sessions without repositioning) on 12 volunteers to ensure intra-examination reproducibility. Nine volunteers underwent two examinations (> 6 months) to ensure inter-examination reproducibility. The regions of interest (ROIs) of the articular disc and retrodiscal tissue were manually selected and calculated. The mean T2 values of the articular disc and retrodiscal tissue were 25.3 ± 3.0 and 30.0 ± 4.1 ms, respectively. T2 mapping showed excellent intra-examination intraclass correlation coefficients (ICCs) for both articular disc (0.923) and retrodiscal tissue (0.951). Very strong correlations (r) were observed in both articular disc (0.928) and retrodiscal tissue (0.953) (*P* < .001). Inter-examination reproducibility also demonstrated that the ICCs were excellent (0.918, 0.935) on both ROIs. T2 values between first and second examinations were strongly correlated (r = 0.921, 0.939) (*P* < .001). In conclusion, T2 mapping seems to be a promising tool for TMJ assessment, regardless of the TMJ condition.

## Introduction

Intra-articular temporomandibular disorders (TMDs) are characterized by positional or morphological changes in musculoskeletal components. Pain, a clicking sound, and restricted movement are common symptoms^1^ that affect the quality of life^2^. To evaluate the temporomandibular joint (TMJ), diagnostic imaging is mandatory as the clinical diagnosis alone could not provide a thorough evaluation^3^.

Magnetic resonance imaging (MRI) is considered to be the gold standard for TMJ examination. Conventional MRI has been used in standard practice, offering a qualitative assessment of disc position and bone evaluation. However, early disc degeneration occurs even before any morphological alterations^4^. Quantitative or biochemical imaging is therefore needed to reveal any ultrastructure that may not appear on conventional MRI. T2 mapping is a quantitative imaging method that has effectively been adopted to detect degenerated cartilage in many other joints by using a T2 relaxation time (also known as a T2 value) as an indirect indicator to reflect the water and collagen contents^5–8^.

Previous studies have revealed a significantly longer T2 relaxation time in retrodiscal tissue^9^, but no significant differences in the articular disc^10^ between volunteers and patients using a 1.5 Tesla (T) MR machine. Moreover, a study predicting intra- and inter-observer reproducibility of T2 mapping in asymptomatic volunteers at 3.0 T was also conducted and proven feasible for TMJ examination^11^. However, to the best of our knowledge, intra- and inter-examination reproducibility of T2 mapping for TMJ assessment at 3.0 T has not been demonstrated.

This study aimed to evaluate whether T2 relaxation times of the articular disc and retrodiscal tissue have enough potential to serve as diagnostic tools using a 3.0 T MR scanner by investigating the intra- and inter-examination reproducibility within the same scanner.

## Results

### Qualitative findings

The qualitative MRI findings of the TMJs in volunteers are summarized in Table 1. Most of the joints (79.4%) were normal superior (NorSup), and five (14.7%) had anterior disc displacement without reduction (ADDWOR). Partial anterior disc displacement with reduction (PADDWR) and anterior disc displacement with reduction (ADDWR) accounted for only 2 joints (5.8%) of the total number of joints. Twenty-nine joints (85.3%) were graded as having none or minimal fluid of joint effusion, and three (8.8%) were considered moderate. Marked and extensive fluid effusions were found in one joint each (2.9%). Osteoarthritis-positive signs were observed in five joints (14.7%). However, bone marrow abnormalities were negative in all joints.

**Table 1:** Volunteer characteristics and qualitative MRI findings.

Variables	n (%)
**Volunteer (cases)**	17
Male	6 (35)
Female	11 (65)
**Age (years)**	
Mean	26.1 ± 2.9
Median	25
Range	23–35
Articular disc position and function (joints)*	
Normal superior	27 (79.4)
PADDWR	1 (2.9)
PADDWOR	0 (0)
ADDWR	1 (2.9)
ADDWOR	5 (14.7)
Joint effusion (joints)*	
None or minimal fluid	29 (85.3)
Moderate fluid	3 (8.8)
Marked fluid	1 (2.9)
Extensive fluid	1 (2.9)
**Osteoarthritis (joints)**	
Negative	29 (85.3)
Positive	5 (14.7)
**Bone marrow abnormality (joints)**	
Negative	34 (100)
Positive	0 (0)

*Percentages may not total 100 because of rounding.

PADDWR, partial anterior disc displacement with reduction; PADDWOR, partial anterior disc displacement without reduction; ADDWR, anterior disc displacement with reduction; ADDWOR, anterior disc displacement without reduction.

### Quantitative findings

The T2 relaxation times are shown in Tables 2 and 3. The overall mean T2 values were 25.3 ± 3.0 ms (range 19.8–33.1 ms) for the articular disc and 30.0 ± 4.1 ms (range 22.6–42.1 ms) for retrodiscal tissue. There were no significant differences in both the articular disc and retrodiscal tissue T2 relaxation times between the first and second intra-examinations (25.2 ± 3.2 and 25.6 ± 3.4 ms; *P* = 0.143, 29.0 ± 4.1 and 28.9 ± 4.4 ms; *P* = 0.582, respectively). Likewise, inter-examination T2 relaxation times of the articular disc were not significantly different from the second examination (26.2 ± 3.1 and 26.5 ± 2.8 ms; *P* = 0.321). Moreover, the retrodiscal tissue demonstrated no significant differences between the two examinations (30.5 ± 4.2 and 30.4 ± 4.8 ms; *P* = 0.77). Joints with positive TMD signs and symptoms (n = 14) had mean T2 values of 26.5 ± 3.3 ms (range 22.4–33.2 ms) and 29.8 ± 4.7 ms (range 22.4–42.1 ms) for the articular disc and retrodiscal tissue, respectively. The mean T2 values of TMD-negative joints (n = 20) were 24.4 ± 2.5 ms (range 19.8–28.1 ms) for the articular disc and 30.1 ± 3.7 ms (range 23.3–37.5 ms) for the retrodiscal tissue. TMD-positive volunteers showed a statistically higher T2 value of the articular disc than TMD-negative volunteers (*P* = 0.037). However, no significant differences were observed when comparing T2 values of retrodiscal tissue (*P* = 0.854) between TMD-positive and TMD-negative volunteers.

**Table 2:** T2 relaxation times of the articular disc.

Volunteers	n (joints)	T2 relaxation time (ms)	*P*-value
Overall	34	25.3 ± 3.0	
Intra-examination (1st)	24	25.2 ± 3.2	
Intra-examination (2nd)	24	25.6 ± 3.4	0.143
Inter-examination (1st)	18	26.2 ± 3.1	
Inter-examination (2nd)	18	26.5 ± 2.8	0.321
TMD-positive	14	26.5 ± 3.3	
TMD-negative	20	24.4 ± 2.5	0.037

TMD, temporomandibular disorders.

**Table 3:** T2 relaxation times of the retrodiscal tissue.

Volunteers	n (joints)	T2 relaxation time (ms)	*P-*value
Overall	34	30.0 ± 4.1	
Intra-examination (1st)	24	29.0 ± 4.1	
Intra-examination (2nd)	24	28.9 ± 4.4	0.582
Inter-examination (1st)	18	30.5 ± 4.2	
Inter-examination (2nd)	18	30.4 ± 4.8	0.77
TMD-positive	14	29.8 ± 4.7	
TMD-negative	20	30.1 ± 3.7	0.854

TMD, temporomandibular disorders.

### Reproducibility analysis

Intra-rater reliability demonstrated that the coefficient of variation (CV%) of T2 relaxation times of five random volunteers ranged from 1.01 to 5.68%, which was considered acceptable. Overall, the intraclass correlation coefficients (ICCs) were good to excellent (0.878–0.993).

The ICCs for intra-examination T2 relaxation times of the articular disc and retrodiscal tissue in 24 joints were 0.923 (95% confidence interval [CI] 0.830, 0.966) and 0.951 (95% CI 0.891, 0.979), respectively. Pearson’s correlations (*r*) were very strong for both the articular disc (0.928) and retrodiscal tissue (0.953) (*P* < 0.001).

The inter-examination ICCs for measuring T2 relaxation times of the articular disc and retrodiscal tissue in 18 joints were 0.918 (95% CI 0.799, 0.968) and 0.935 (95% CI 0.835, 0.975), respectively. The correlation coefficients (*r*) were very strong for the articular disc (0.921) and retrodiscal tissue (0.939) (*P* < 0.001).

**Figure 1:**
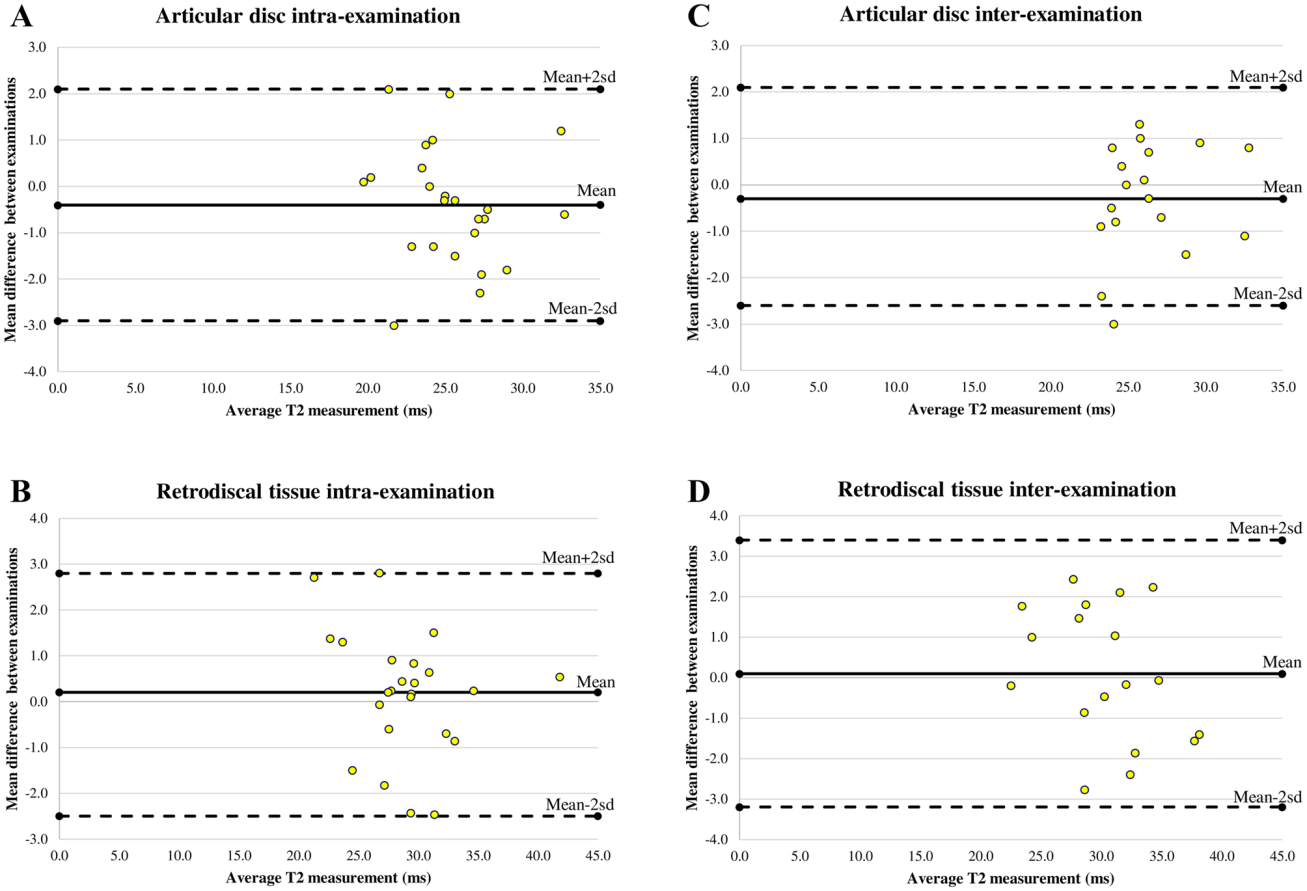
Bland–Altman plots for (**A**) intra-examination reproducibility of the articular disc, (**B**) intra-examination reproducibility of retrodiscal tissue, (**C**) inter-examination reproducibility of the articular disc, (**D**) inter-examination reproducibility of retrodiscal tissue.

The Bland–Altman plots of the difference between the two examinations and average T2 measurements were constructed with lower and upper limits of agreement (mean differences ± 2 standard deviation [SD]). The intra-examination limits of agreement for the articular disc and retrodiscal tissue were − 0.4 (95% CI − 2.9, 2.1) and 0.2 (95% CI − 2.5, 2.8), respectively (Fig. 1A, B). The lower and upper limits of agreement for inter-examination of the articular disc were − 0.3 (95% CI − 2.6, 2.1), and of the retrodiscal tissue were 0.1 (95% CI − 3.2, 3.4) (Fig. 1C, D).

### Visual analog scale (VAS) evaluation

The assessment of VAS score for inter-examination reproducibility demonstrated no statistical differences between the two examinations for all aspects, including VAS at rest (*P* = 0.89), during jaw movement (*P* = 0.128), during meals (*P* = 0.141), and daily life interference (*P* = 0.314). According to the regression analysis, the VAS score could not predict the T2 relaxation times of both the articular disc and retrodiscal tissue (*P* = 0.397, 0.69, respectively).

## Discussion

This is the first study to report intra- and inter-examination reproducibility of TMJ assessment at 3.0 T. The main findings of this study were as follows: (1) Intra-examination reproducibility revealed excellent ICCs and very strong correlations for both the articular disc and retrodiscal tissue. The scatterplots of the Bland–Altman plot lie within the upper and lower limits (mean difference ± 2SD). (2) Inter-examination reproducibility also suggested excellent ICCs and very strong correlations for all the regions of interest (ROIs). Good agreement was observed in the Bland–Altman plots.

T2 values of the articular disc were reported in previous studies showing both similar (26.9 ± 3.7, 29.3 ± 3.8, and 25.19 ± 1.15 ms)^10–12^ and much longer values (40.21 ± 2.95 ms)^13^ in healthy volunteers compared to our TMD-negative volunteers. In those studies, MRI examinations were performed using TMJ surface coils. However, the scans at our institution were carried out using a head coil because it improves the overall image quality and accuracy for the articular disc and bilaminar zone^14^. Sun et al.^15^ stated that both coils could be used for TMJ MRI examination. However, they still suggested using a TMJ surface coil for conventional imaging and a head coil for dynamic imaging owing to its higher signal-to-noise ratio (SNR) values. Moreover, we used a higher magnetic field (3.0 T) than other studies. Not only has it been shown to enhance structural analysis in healthy TMJs^16^, but it also yields a superior joint definition without increasing examination time^16,17^ and an improvement in SNR^18^.

Previous studies with comparable T2 values included volunteers with age groups similar to ours^10–12^. However, the study with elongated T2 relaxation time did not provide age group information^13^. This could be relevant as Kakimoto et al.^10^ reported that older patients with TMD demonstrated a significantly longer T2 relaxation time. In contrast, a study carried out in adolescents (age range 7–20 years) showed a surprisingly high T2 relaxation time (39.3 ± 5.6 ms)^19^.

In our study, volunteers with positive signs and symptoms of TMD had a significantly longer articular disc T2 relaxation time than TMD-negative volunteers. However, no differences were detected in the retrodiscal tissue. Therefore, it is difficult to confirm this relationship without further investigation in larger groups. Several studies have reported results ranging from no differences (of the articular disc)^10,20^ to significant differences showing higher T2 values than normal volunteers (of the articular disc^12^ and retrodiscal tissue^9^), which are in agreement with our results. However, the opposite trend was observed by Zhao et al.^19^, who demonstrated a statistically significant decrease in T2 values of the articular disc when disc displacement was encountered.

Differences observed between many studies are likely due to the anatomical complexity of the temporomandibular complex, as they are closely packed within small areas, which can be relatively difficult to locate precisely. T2 mapping of the TMJ tends to be more susceptible to influence from neighboring tissues and structures than in other larger joints. T2 values of the articular disc can arise from the effect of nearby joint effusion, similar to a previously manifested result in the knee cartilage^20,21^. Owing to the complexity of the joint, the visibility of retrodiscal tissue has been reported to be at 73.6% for the normal disc. It was even lower for those who had disc displacement with or without reduction at 43.5%^22^. These could greatly impact the ROI selection process and the outcome of T2 relaxation time to be vastly different among studies.

In TMD-related patients, pain is the most prevalent finding among other factors. VAS scores were then considered in the data analysis to reduce the possible impact on T2 relaxation time. In this study, a comparison of the VAS scores between the two examinations showed no significant differences. Therefore, we can confidently expect that the results for inter-examination reproducibility were not affected by variations in the pain levels of the volunteers.

The key strengths of the current study are the use of a head coil at 3.0 T for superior MR image quality, with excellent ICCs and very strong correlations. We additionally confirmed good agreement with the Bland–Altman analysis. The scope of this study was limited by the small number of volunteers included in the T2 relaxation time examination. In addition, the generalizability was challenging to interpret, as we recruited volunteers regardless of TMD diagnosis. However, we categorized them into TMD-positive and TMD-negative groups based on clinical symptoms and MRI information. Although they differed in number, the proportion was still acceptable for statistical analysis. We recommend that future research be undertaken under the following settings: (1) recruitment of more subjects, (2) inclusion of both TMD and TMD-free volunteers, and (3) finding a representative value for TMD diagnosis.

## Methods

### Volunteers

The study was conducted in accordance with the guidelines of the Declaration of Helsinki and approved by the Institutional Review Board of Hiroshima University Graduate School (E-1059, December 26, 2017). Prior to the examinations, details of the procedure were carefully explained, and written informed consent was obtained from all volunteers.

**Figure 2:**
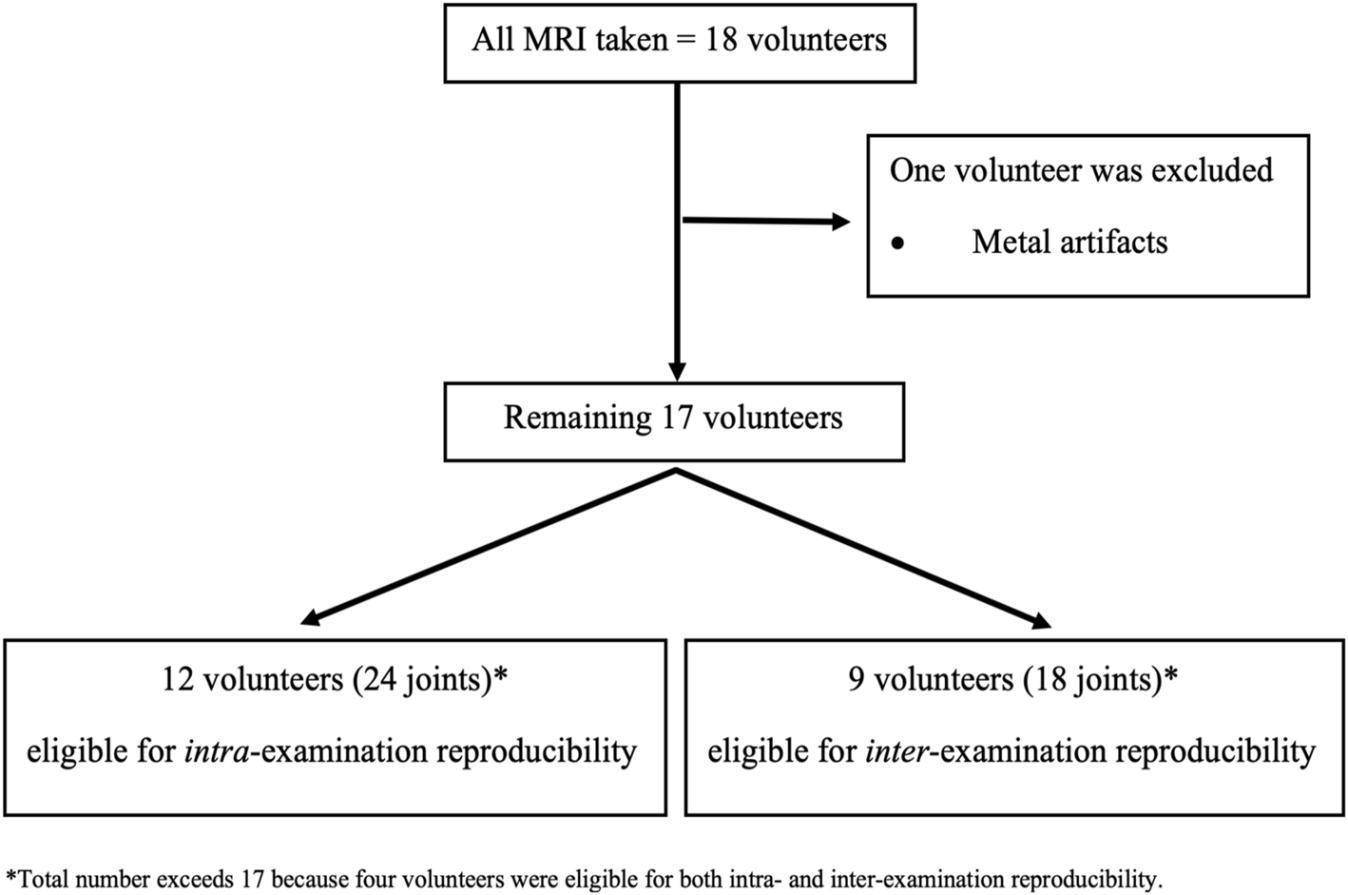
Volunteer selection and distribution flowchart.

From 2018 to 2021, 18 volunteers were recruited regardless of the signs and symptoms of TMD. One volunteer was excluded because of metal artifacts. Therefore, 17 volunteers were included in this study. Twelve volunteers were included for intra-examination reproducibility, and nine for inter-examination reproducibility (four volunteers underwent both intra- and inter-examination reproducibility tests), as shown in Fig. 2. Of the 17 volunteers, 11 (65%) were female, and six (35%) were male, with a mean age of 26.1 ± 2.9 years (age range, 23–35 years).

### MRI data acquisition

All volunteers underwent MR examinations of their TMJs using a 3.0 T MR scanner (Ingenia CX 3.0 T scanner, Philips, The Netherlands) equipped with a ds head 32ch coil. The imaging protocol consisted of oblique sagittal and coronal proton density (PD)-weighted turbo spin-echo (TSE) and fat-suppressed sagittal T2WI TSE sequences with the mouth closed. Open mouth positions were obtained using a sagittal PD-weighted TSE sequence. T2 mapping sequences were performed to measure the T2 relaxation times of the TMJ and surrounding structures using six-echo TSE at the closed-mouth position. Six turbo (TSE) factors were used, and the echo times were as follows: 16.0, 24.0, 32.0, 40.0, 48.0, 56.0 ms. Compressed sensing (CS) combined with sensitivity encoding (SENSE) or compressed SENSE (CS SENSE) was applied to some patients to reduce the scan time and improve spatial resolution. All scan parameters are listed in Table 4. To acquire data for intra-examination (in-scan) reproducibility, the T2 mapping sequences were repeated without repositioning the volunteers (> 5 min apart between each scan). On the other hand, inter-examination (scan-rescan) reproducibility measurements were performed by undergoing two separate T2 mapping examinations approximately six months apart.

**Table 4:** Scan parameters.

	PD oblique sagittal and coronal	Fat-suppressed T2WI sagittal	PD sagittal open mouth position	T2 mapping at oblique sagittal
FOV (mm × mm)	120 × 120	120 × 120	120 × 120	120 × 120
Acquisition matrix	256 × 166	256 × 186	224 × 156	224 × 135
Slices	26	26	26	12
Slice thickness (mm)	3	3	3	4
Slice gap (mm)	0	0	0	0.5
TR (ms)	3000	3000	3000	2100
TE (ms)	8	60	8	16.0–56.0
TSE factor	7	14	8	6
Flip angle (degree)	90	90	90	90
Number of averages	1	1	1	1
Scan time (min)	1:54 (CS SENSE) or 3:06	1:39 (CS SENSE) or 2:42	1:18 (CS SENSE) or 1:54	6:20
Percent phase FOV (%)	100	100	100	100
Percent sampling (%)	64.7	72.6	69.9	60.2
Pixel bandwidth	614	436	698	436
MR acquisition type	2D	2D	2D	2D
Pixel spacing	0.234 × 0.234	0.234 × 0.234	0.234 × 0.234	0.234 × 0.234

FOV, a field of view; TR, repetition time; TE, echo time; TSE, turbo spin-echo; PD, proton density; CS SENSE, compressed SENSE.

### MRI characteristic evaluation

All MR images were morphologically assessed in many aspects, including disc position, joint effusion, osteoarthritis, and bone abnormalities^10^. Disc dislocations were evaluated and classified into five categories according to Tasaki et al.^23^, including NorSup, PADDWR, partial anterior disc displacement without reduction (PADDWOR), ADDWR, and ADDWOR. Joint effusion was categorized using a grading system by Larheim et al.^24^ into four groups: (1) non-observed or minimal fluid, (2) moderate fluid, (3) marked fluid, and (4) extensive fluid in the closed-mouth position. When bone changes, such as osteophytes or erosion, are present, the joint will be considered osteoarthritis-positive. However, in the absence of both signs, the joint will be osteoarthritis-negative, as suggested by Kirk^25^. The presence of edema or osteonecrosis in the TMJ was classified as positive for bone marrow abnormalities described by Larheim et al.^26^ These features, together with pain scores (VAS score > 4), were later taken into consideration when grouping volunteers. Those with TMD-related signs and symptoms will be labeled as TMD-positive, and TMD-negative will represent asymptomatic volunteers without any of the signs listed above.

### Measuring T2 relaxation times

**Figure 3:**
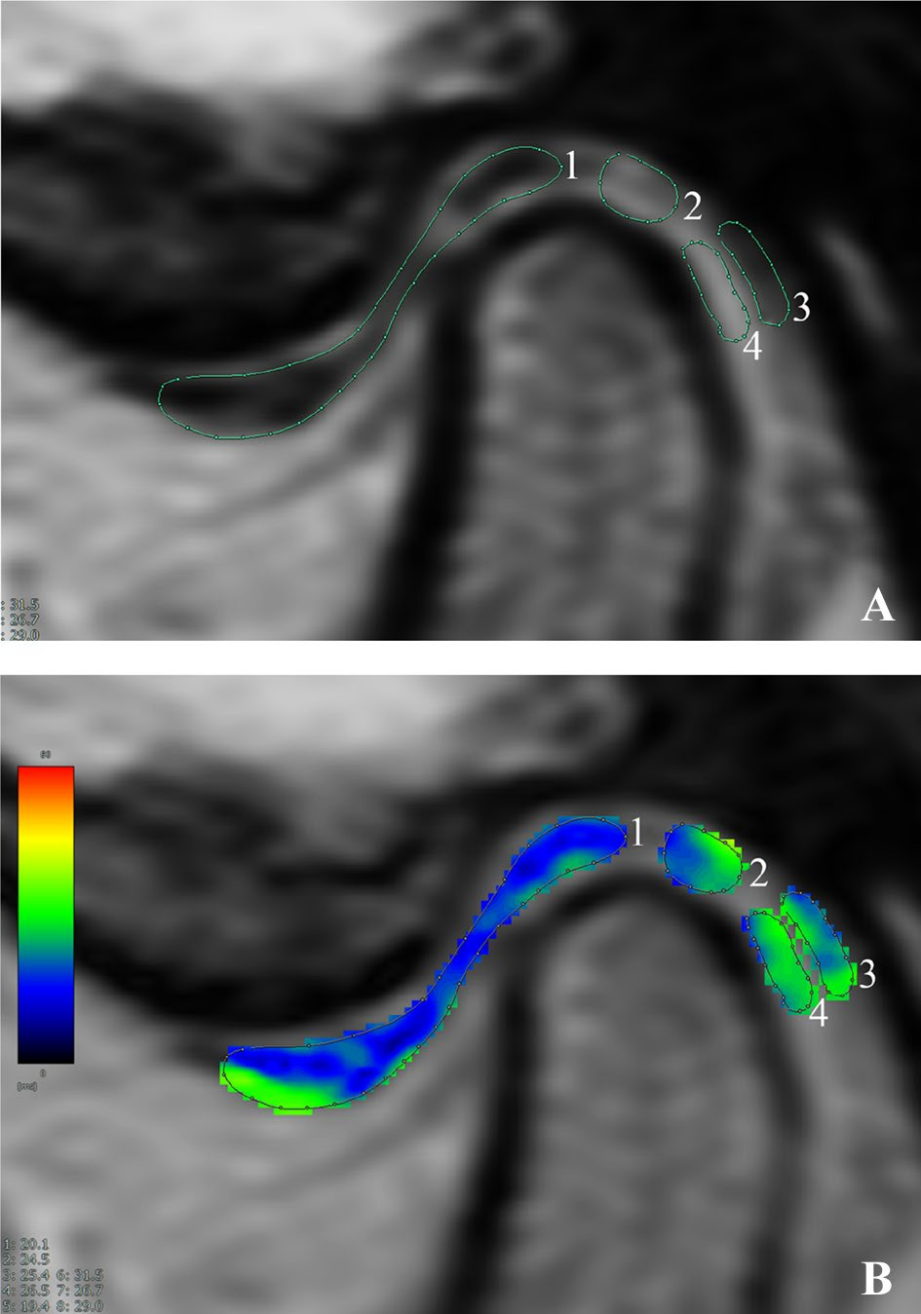
MR parasagittal images of the temporomandibular joint in closed-mouth position at 3.0 T, used for T2 relaxation time measurement. (**A**) ROI selection for the articular disc (1), bilaminar zone (2), superior and inferior lamina (3, 4). (**B**) The T2 relaxation times of ROIs integrated with a color-coded map displaying values from 0 (dark blue) to 60 (red) ms.

All images were transferred to a dedicated workstation (SYNAPSE VINCENT; Fujifilm Medical, Tokyo, Japan) to assess the T2 relaxation times. The ROIs of the entire articular disc and retrodiscal tissue (see Fig. 3), previously conducted by Kakimoto et al.^9,10^, were manually selected (by P.W.) on images with the most suitable contrast. The T2 relaxation time of the retrodiscal tissue was obtained from the average of three ROIs, including the bilaminar zone abutting the articular disc, superior lamina, and inferior lamina.

### VAS evaluation

Along with the inter-examination reproducibility test, VAS scores were collected at both the first scan and rescan, including VAS at rest, VAS during jaw movement, VAS during meals, and VAS of daily life interference. A hundred millimeter-scale was used, and the score ranged from zero as no pain to a hundred as severe intolerable pain. Volunteers decided on their current pain level on the examination day and marked it on the scale. The scores were later assessed using a ruler to quantify the VAS scores. VAS categories from Jensen et al.^27^ were adopted for simpler interpretation as follows: 0–4; no pain, 5–44; mild pain, 45–74; moderate pain, 75–100; severe pain.

### Statistical analysis

All statistical analyses were performed using IBM SPSS Statistics version 28.0 (SPSS Inc., Chicago, IL, USA). MRIs from five volunteers were randomly selected to assess the reliability of T2 relaxation time measurements. ROIs on the TMJ disc and retrodiscal tissue were manually delineated, and the procedure was repeated ten times with a two to three day interval between each session. To assess intra-rater reliability, CV%, ICC, and 95% CI were calculated based on a single rater, absolute agreement, and two-way mixed-effects model or ICC (2,1). A CV% of less than 10% was considered acceptable, and mean estimations along with 95% CI were reported for each ICC. The ICC agreement was interpreted according to Koo & Li, 2016 as follows: 0.00–0.50, poor; 0.50–0.74, moderate; 0.75–0.90, good, and 0.90–1.00, excellent^28^.

For intra- and inter-examination reproducibility, the Shapiro–Wilk test was used to determine whether the data were normally distributed. Both sets of data appeared to be normally distributed. Paired *t-*tests were used to compare the T2 relaxation times of the TMJ disc and retrodiscal tissue between the first and second examinations. Pearson’s correlation coefficient (*r*) and ICC (2,1) were calculated for both assessments. A guideline by Chan^29^ on the interpretation of Pearson’s correlation coefficient was integrated to determine the strength of the relationship as follows: less than 0.3, poor; 0.3–0.5, fair; 0.6 up to 0.8, moderately strong; and at least 0.8, very strong. Bland–Altman plots were applied to investigate the limits of agreement using the mean and standard deviation between the two measurements. Visual estimation was achieved by plotting the differences against the mean. It was suggested that 95% of scatterplot should lie within upper and lower bound or mean difference ± 2SD^30^. The independent samples *t-*test was performed to compare the T2 relaxation times of the articular disc and retrodiscal tissue between TMD-positive and TMD-negative volunteers.

VAS scores were compared in those eligible for inter-examination reproducibility. However, the data were not normally distributed. A nonparametric Wilcoxon signed-rank test was used. Additionally, regression analysis was performed to determine any relationship between the VAS score and T2 relaxation times of the articular disc and retrodiscal tissue. Statistical significance was set at *P* < 0.05.

## Conclusions

In conclusion, T2 mapping at 3.0 T is a reproducible method for quantifying the biochemical composition of the articular disc and retrodiscal tissue in intra- and inter-examinations. TMD-positive volunteers tended to have longer T2 relaxation times in the articular disc than those without TMD. This suggests that T2 mapping might potentially be a diagnostic tool for early TMD. However, a more extensive study should be conducted to reduce the potential influences caused by the high sensitivity of T2 mapping and establish reliable representatives and a possible cutoff value for TMD diagnosis. Then, we would be able to confidently verify the results and start using T2 relaxation times in daily clinical practice.

## Data Availability

The datasets used and analyzed during the current study are available from the corresponding author on reasonable request.
